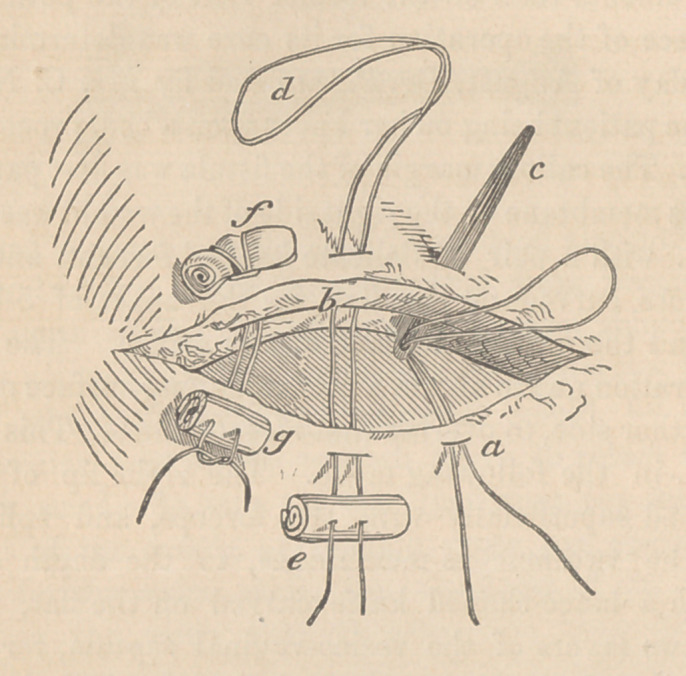# Cure of a Vesico-Vaginal Fistula, by an Operation by Professor Pancoast

**Published:** 1851-10

**Authors:** C. D. Meigs


### *Cure of a Vesico-Vaginal Fistula, by an operation by* Professor Pancoast. By C. D. Meigs, M. D.

In the month of October, 1848, a lady was delivered, by mutilation of the fœtus, after a most painful and protracted labor ; the child having presented by the shoulder. I am not informed further than this, as to the particulars of the case. After recovering her composure from the greatest fatigue and exhaustion, she found herself always wet, and felt no desire at any time to pass the urine. By slow degrees she regained her health, with the exception that she had constant *stillicidium urinæ*, which occasioned painful inflammation and excoriation of the external privities and inner sides of the thighs ; and she was annoyed by the urinous odor, which it was impossible to suppress.

After a length of time, she was informed that the cause of her distress depended upon a laceration affecting the urethra, so nigh to the neck of the bladder as to prevent the *sphincter vesicæ* from controlling the escape of the urine. During many consecutive months, attempts were made, by cauterization with nitrate of silver, to cause the aperture to close—which was in vain.

About six months ago, she came to me for advice in the case, when, together with Prof. Pancoast, I found the circumstances as I shall attempt by means of the figure to explain:

(*a*) Is the bladder of urine seen from its under surface. *b*, *b*, a catheter in the urethra; a part of the catheter is visible in the fissure, (*d*) just below the orifice of the bladder. The excessive resistance of the perineum made it then impossible to get at the fissure with instruments fit to pare off the edges, or with needles to close the gap. It is probable that the highly irritated state of the parts, brought on by the continual flowing of urine over them, may have rendered access to the fissure out of the question. Be that as it may, it was not then deemed possible to operate on the case.

My colleague, Professor Pancoast, finding it too difficult, with various methods of exploration, to bring the injured part within the reach of his instruments, concurred with me in advising her to postpone any definitive attempts to cure by operation until the fall of the year; with a view to her using in the mean time, a certain precaution, by which we hoped to render the case more manageable after the lapse of a few months. The precaution consisted in the use of a tube which I have described in the Transactions of the Philadelphia College of Physicians, and which I caused to be made for her, and which was shaped as follows, viz: it was a silver gilt canula, 2¼ inches long, of a spindle shape, with a small funnel adapted to one end of the spindle, and a perforated disk to the other end, as in the annexed figure:

I supposed that such a canula, if introduced along the urethra, quite to the bladder, would be prevented from passing wholly into that organ by the small triangular disk or shield, (*a*) that the meatus urinæ would firmly contract on the narrow part of the canula, (*b*) whilst the bulging portion (*c*) would, by distending a less muscular part of the urethra, serve to retain the instrument more surely in situ. I designed that the small cylindrical part of the canula (*d*) should cross the opening as an acqueduct for the urine, while the smallness of its size would enable it at the same time to allow the fissure to close the more readily, *par defaut d'extension.* The funnel (*e*) at the end of the canula, was to pass just within the orifice of the bladder and no farther, in order to collect the urine, which was thus to be conducted out upon sponges placed in front of the outer shield, which was properly adapted to fill up the triangular space of the vestibulum.

Such an instrument placed in the urethra, would not be thrust forth except the patient should make violent efforts to expel it. We hoped that by thus preventing the continual flow of the urine through the lacerated part, it might tend to close up so much as to render an operation practicable and successful; and I explained to her that such a conductor for the urine, if it could be worn for a long time, would perhaps serve at last as a cure, without resort to operation by the knife. She was begged to persist in the trial for several months—but came back again in about 150 days—being impatient of her sufferings.

We thought great good was effected by the use of the canula, which she used during the greater part of the time, being, however, during her absence from town, annoyed to that degree, as to be sometimes obliged to suspend the use of it—which was reasonably to be expected.

On a re-examination of the fistula in connection with Dr. Pancoast, on 13th of August, we found it much more easily reached with instruments than on the former visit of the patient, and the performance of the operation for its cure was determined on. On the 16th day of August, Dr. P., assisted by Mr. C. Neff, and by myself, the patient being on her knees upon a bed, proceeded to the operation. The callous margin of the fistula was first pared away—the mucous membrane on the right side of the wound was then seized by Dr. P. with a pair of delicate hooked forceps, and detached with scissors curved on the flat, for the space of 5-8ths of an inch around the semicumference of the orifice. The next stage of the operation was to obtain a corresponding surface on the opposite or further side, to overlap that already made. This was gained by Dr. P. in the following mode. The right lip of the orifice was grasped superficially with the forceps, and split mid-way between the two mucous membranes, to the depth of half an inch, with a lance-shaped knife curved on the flat, making, as it were, two layers of the vesico-vaginal septum, for the space of nearly an inch in length and half an inch in depth, as described in the figure:

The lance-shaped knife very readily made a wound by thrusting it into the bladder edgewise, as I have endeavored to show by the representation annexed. It is evident, if the tissues can be firmly held by the hook forceps, as in the diagram, that the lance-shaped knife can very readily be thrust into the edge or lip of the wound, so as to split it as much as could be desired, and that such a wound would be completely fresh.

Let (*a*) be the revived surface of the lower lip of the fissure. Let (*b*) be the split and gaping wound made by means of the lance-shaped knife in the edge of the upper lip of the fissure; (*c*) is a short flat needle, with the eye in the upper part, and armed with a double thread or ligature. It is passing out at the upper part of the split lip, and from thence is to be drawn forth by means of a dressing forceps; (*d*) may represent the same ligature from which the needle has been removed; (*e*) is a piece of adhesive plaster made into a hard roll like bougie, and hammered out flat; with two small holes drilled through it to admit of threading the ligatures through them; (*f*) is the ligature (*d*) adjusted with a loop over a small quill, composed of adhesive plaster rolled into a small cylinder or bougie; (*g*) is the lower quill, (*e*)

Now it is clear that if the lower lip (*a*) can be freshened with with scissors curved on the flat, (a very possible operation,) and if the upper lip (*b*) can be split or divided by a lance-shaped knife; and if *g* and *f* can, by pushing *g* forwards to *f*, be closely approximated, the opposite surfaces *a* and *b* may be brought into effectual and protracted contact, and thereupon, if adhesive inflammation follows, the patient will be cured.

From the success of the operation above related, I am led to believe, that few of those unfortunate women who would otherwise be doomed to a life of disgusting misery after laceration of the bladder or urethra, need be left in hopeless incurable distress; and it is with a view to communicate to the brethren in Medicine everywhere, the knowledge of this simple and effectual process, that I have drawn up these notes.

I should commit an act of injustice, however, if I should stop here without making an explanation due to a Surgeon, who deserves, and indeed has my highest respect. I speak of Dr. Sims, of Montgomery, Alabama.

In a conversation with him in the end of July, 1851, he informed me of his many successful operations in vesico-vaginal fistula—a success which in my humble opinion entitles him to the praise and gratitude of our whole profession, which is always advanced by the ingenious and rational improvements made by such individuals, and which constitute a real progress of the Art. Dr. Sims communicated to me the fact, that if the patient, laboring under this accident, be placed in a certain position, the injured parts may be readily brought not only in sight, but so exposed as to render them easily accessible to the knife, the scissors, &c., &c.

His own judgment prompted the discovery of this method, which consists in placing the patient on her knees with the ossa femoris standing in a vertical position while the summit of the thorax touches the table or bed on which she is placed.

If such a posture be maintained for a few minutes, the fall of the abdominal and pelvic viscera downwards toward the diaphragm, allows the os externum to relax, to open, and at last to offer so wide an aperture, that the cervix uteri may be clearly seen as in a metroscope. In the meantime, the fall of the viscera continuing, the walls of the vagina, to avoid the tendency to vacuum caused by the retreat of the pelvic contents toward the diaphragm, tend to expand, and like a balloon leave a spherical or orbicular cavity, as if the vagina was filled with a child's head, whereas it is filled with air.

This discovery is in the highest degree applicable to the uses of curative surgery in vesico-vagina fistula.

The great experience and success of Dr. Sims will enable him, it is hoped, soon to give his results to the profession. In the meantime, I trust I shall give no offence while in describing the several steps of the operation on my patient, if I take this early occasion to thank Dr. Sims for his information, and to acknowledge how much I am his debtor therefor.

From the moment of completing the operation above described, not a drop of urine escaped through the fissure. She wore the spindle-shaped canula during the ten or twelve days following and then removed it. The ligatures were easily cut away and picked out with dressing forceps, and we had the happiness to see the patient completely relieved of the effects of an accident, which nothing but a religious resignation could have enabled her to bear with patience, or to regard life otherwise than as a distressing burthen, to be borne only because it was God's will that she should patiently endure its weight. The cure was rapid and complete.

## Figures and Tables

**Figure f1:**
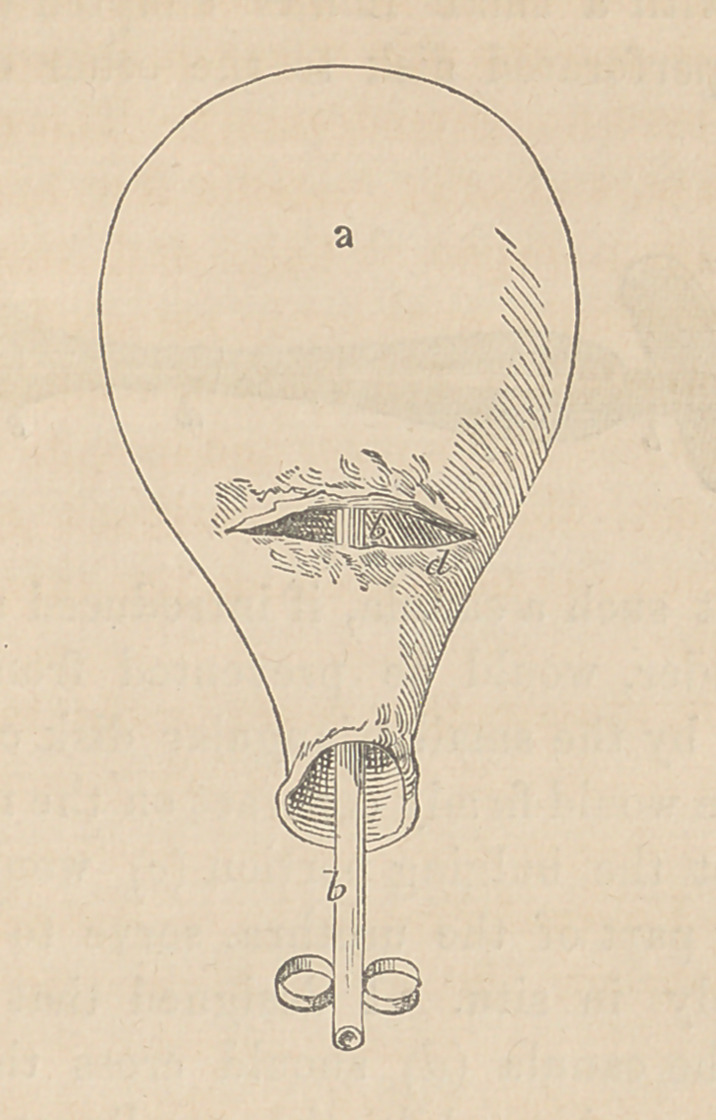


**Figure f2:**
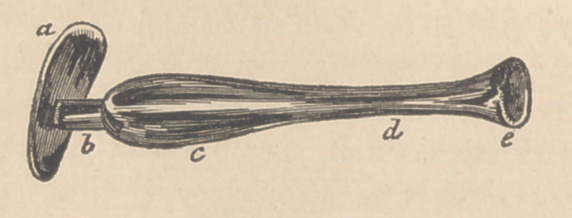


**Figure f3:**
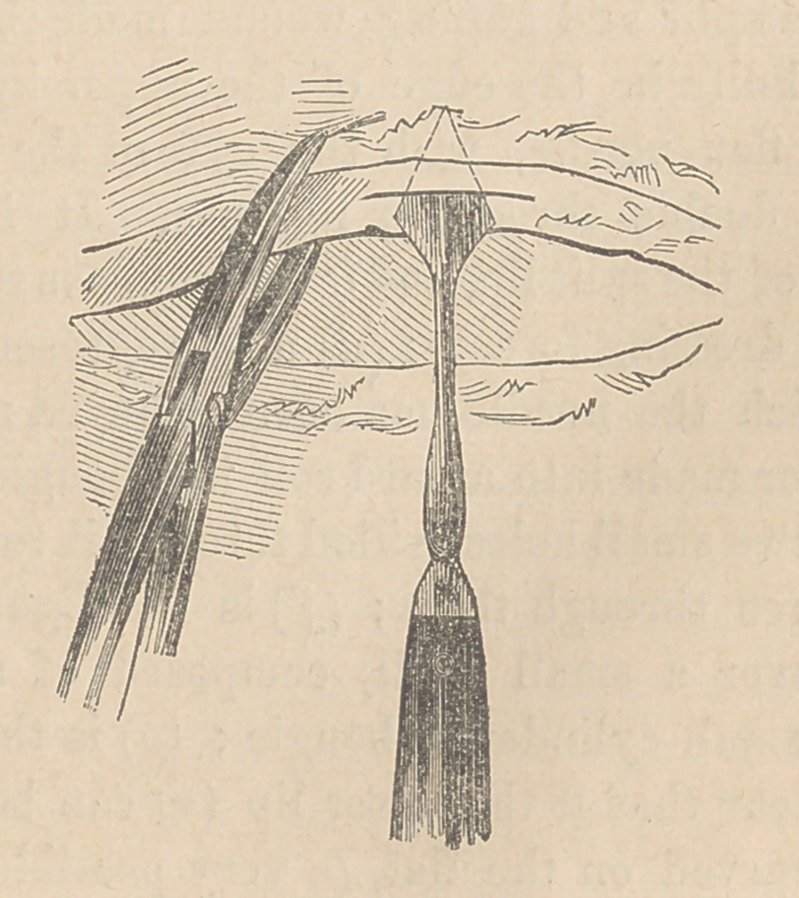


**Figure f4:**